# Publication of Original Research in Urologic Journals – A Neglected Orphan?

**DOI:** 10.1371/journal.pone.0052420

**Published:** 2012-12-19

**Authors:** Jens Mani, Jasmina Makarević, Eva Juengel, Hanns Ackermann, Karen Nelson, Axel Haferkamp, Roman A. Blaheta

**Affiliations:** 1 Department of Urology, Johann Wolfgang Goethe-University, Frankfurt am Main, Germany; 2 Institute of Biostatistics, Johann Wolfgang Goethe-University, Frankfurt am Main, Germany; 3 Department of Vascular and Endovascular Surgery, Johann Wolfgang Goethe-University, Frankfurt am Main, Germany; Innsbruck Medical University, Austria

## Abstract

The pathophysiologic mechanisms behind urologic disease are increasingly being elucidated. The object of this investigation was to evaluate the publication policies of urologic journals during a period of progressively better understanding and management of urologic disease. Based on the ISI Web of Knowledge Journal Citation Reports and the PubMed database, the number and percentage of original experimental, original clinical, review or commentarial articles published between 2002–2010 in six leading urologic journals were analyzed. “British Journal of Urology International”, “European Urology”, “Urologic Oncology-Seminars and Original Investigations” (“Urologic Oncology”), “Urology”, “The Journal of Urology”, and “World Journal of Urology” were chosen, because these journals publish articles in all four categories. The publication policies of the six journals were very heterogeneous during the time period from 2002 to 2010. The percentage of original experimental and original clinical articles, related to all categories, remained the same in “British Journal of Urology International”, “Urologic Oncology”, “Urology” and “The Journal of Urology”. The percentage of experimental reports in “World Journal of Urology” between 2002–2010 significantly increased from 10 to 20%. A distinct elevation in the percentage of commentarial articles accompanied by a reduction of clinical articles became evident in “European Urology” which significantly correlated with a large increase in the journal’s impact factor. No clearly superior policy could be identified with regard to a general increase in the impact factors from all the journals. The publication policy of urologic journals does not expressly reflect the increase in scientific knowledge, which has occurred over the period 2002–2010. One way of increasing the exposure of urologists to research and expand the interface between experimental and clinical research, would be to enlarge the percentage of experimental articles published. There is no indication that such policy would be detrimental to a journal’s impact factor.

## Introduction

Experimental research in the field of urology has become increasingly productive as scientists explore the molecular background of urologic disease. Various scholarships provided by the international urological society support not only understanding of urologic patho-physiology but also encourage translation of novel discoveries into new ideas for prevention, diagnosis and treatment of urologic disorders. The pharmaceutical industry concentrates on applied research, making university laboratories dealing with in vitro and in vivo models attractive business partners to conduct experimental research projects. A final reason for the importance of experimental urologic research is that expertise is a prerequisite to establishing national and international collaboration.

Having established the importance of experimental research in urology one might assume that urologic journals reflect the trend and are publishing an increasing number of papers dealing with experimental research. In fact, however, there are indications that research is not reaching the majority of urologists. Only one-third of the US academic medical centers exclusively conduct basic science research [1]. Eberli and Atala have criticized that only a minority of urologists are currently exposed to significant research experience [2]. Olumi and Dewolf have asked for the support of urology physicians with scientific expertise to drive the emergence of clinically relevant therapies and to foster critical thinking about feasible therapeutic possibilities [3]. Without doubt, increasing urologists’ exposure to scientific research would be beneficial.

Communication between experimental and clinical urologic scientists is of great importance [4] and urologic journals can serve as a key platform where knowledge exchange takes place. This investigation evaluates the publication policies of six leading international urologic journals during the last 9 years.

## Methods

### Journals

Based on the subject category “Urology and Nephrology” in the ISI Web of Knowledge Journal Citation Reports, six leading urologic journals were selected: “British Journal of Urology International”, “European Urology”, “Urologic Oncology-Seminars and Original Investigations” (“Urologic Oncology”), “Urology”, “The Journal of Urology”, and “World Journal of Urology”. The selected journals publish both original clinical as well as original experimental articles in the field of urology and additionally publish reviews and commentary articles. A quantitative longitudinal study was conducted to examine the number of published articles in each journal in the categories experimental, clinical, review and commentary over a 9 year period (2002–2010).

### Analysis Strategy

Articles from each journal were categorized from 2002 to 2010 in 2 year intervals (2002, 2004, 2006, 2008, 2010) using the PubMed database. Each journal was analyzed by year and volume. Articles in supplementary magazines were excluded. The following categories were defined:

Experimental: original experimental research articles

Clinical: original clinical research articles

Review: review articles

Commentarial: articles not fitting in the 3 previous groups: comments, letters, editorials, errata, notices of retraction and technical reports

The number of articles in each category was determined and expressed as a percentage of the total number of articles in the journal. Possible correlations between the percentage of a particular article category and the journal’s impact factor were examined.

To additionally explore whether the publication aims of the journals have changed over the years, the written information for authors as well as the aims and scopes of the journals between 2002 and 2010 were compared.

### Statistics

Statistical analysis was performed with Pearson correlation and regression coefficient and Neumann’s trend analysis. The trend analysis was carried out from 2002 and from 2004. P values <0.05 were considered significant. Evaluation was conducted using the BiAS for windows statistical software program (Version 9.12).

## Results

To evaluate whether urologic journals have changed their publication policies during the past decade it was necessary to select those magazines that cover a broad field of urology and publish the four types of articles (original clinical, original experimental, reviews, commentaries). From 73 journals listed in the subject category “Urology and Nephrology” in the ISI Web of Knowledge Journal Citation Reports only 6 journals fulfilled these criteria. The overall number of articles appearing in all selected journals increased approximately 30% over the years from 2002–2010, except in “The Journal of Urology” where the number of articles remained about the same. As the total number of articles in all four categories increased over the period 2002 to 2010 (from 2,980 to 3,945), so did the number of original experimental articles (from 283 to 370; +30.7%) as well as the number of original clinical articles (from 1272 to 1493; +17.4%). Related to each single journal, the number of original experimental articles in 2002 versus 2010 was as follows: 50 versus 63; +26% (“British Journal of Urology International”), 23 versus 28; +22% (“European Urology”), 158 versus 109; −31% (“The Journal of Urology”), 13 versus 24; +85% (“Urologic Oncology”), 32 versus 121; +278% (“Urology”) and 7 versus 25; +257% (“World Journal of Urology”). The percentage of articles in the experimental category was about 10% of all the articles compiled from all the journals (2002∶11.52+/−6.79%, 2004∶8.74+/−3.24%, 2006∶7.71+/−3.63%, 2008∶9.35+/−5.63%, 2010∶11.39+/−5.44%).

**Figure 1:**
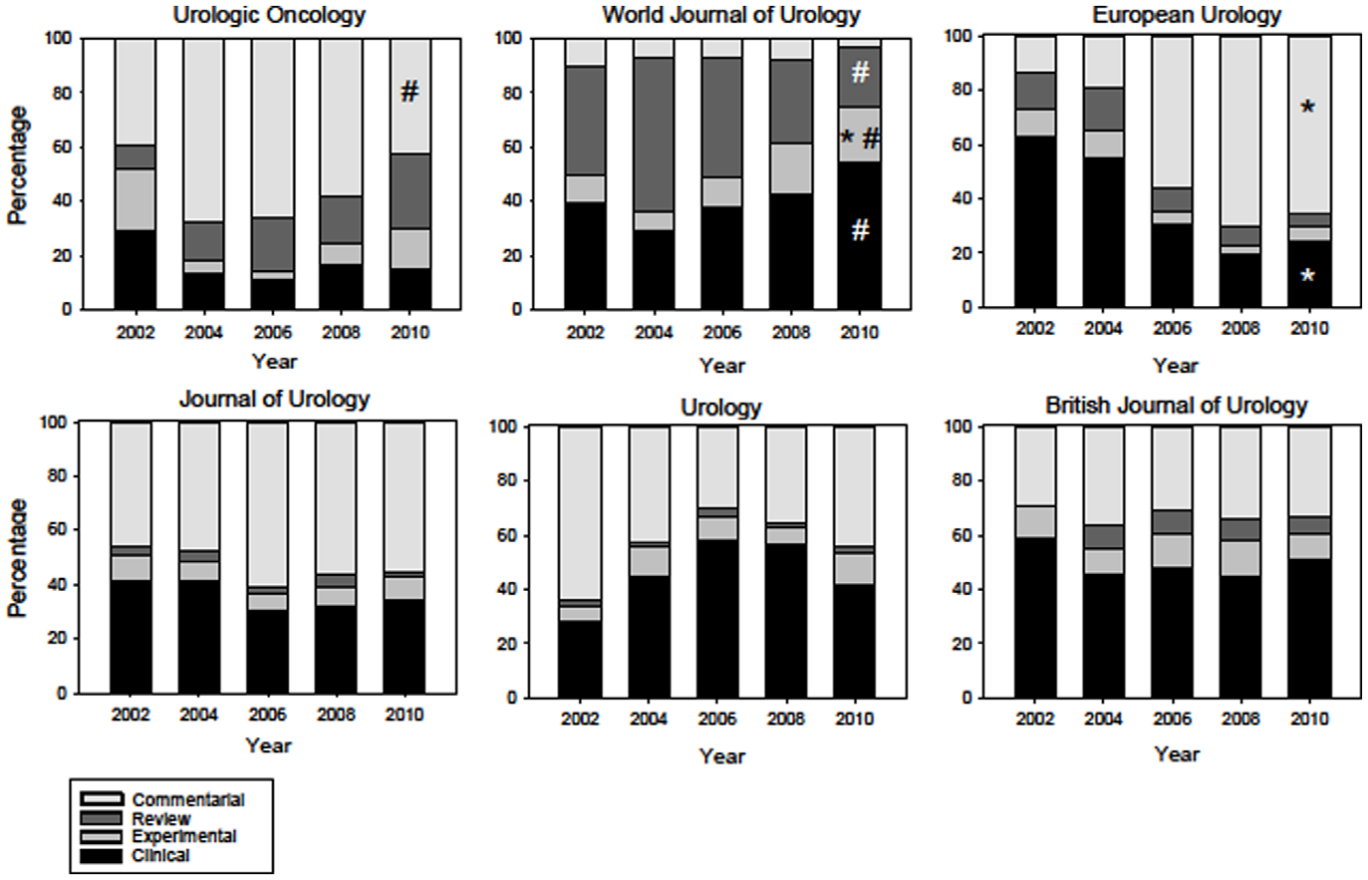
Percent categorical changes in six urologic journals from 2002–2010. ★ = significant Neumann trend from 2002. # = significant Neumann trend from 2004.

Significant trends in the percentage of articles in some of the four categories, experimental, clinical, review and commentary were apparent in three journals: “World Journal of Urology”, “European Urology” and “Urologic Oncology” (Figure 1). In “European Urology” clinical articles were reduced from approximately 60% of all articles in 2002 to 20% in 2010. At the same time commentarial articles increased from approximately 15% to 65%. In “World Journal of Urology” a significant trend was apparent in the percentage of experimental papers, which increased from about 10% in 2002 to 20% in 2010. From 2004 to 2010 trends were apparent in the journals “Urologic Oncology” and “World Journal of Urology”, with respect to commentarial and clinical articles. In “Urologic Oncology” commentarial articles significantly decreased from approximately 70 to 50%. In “World Journal of Urology”, from 2004 to 2010, the percentage of clinical articles significantly increased from about 30 to 50% and reviews decreased from about 60% to 20%. Comparison of the journals’ aims and scopes between 2002 and 2010 revealed no differences with respect to “The Journal of Urology”, “Urology”, “British Journal of Urology International”, “Urologic Oncology” and “World Journal of Urology”. Aims and Scopes of “European Urology” were modified in as much as the journal now publishes “peer-reviewed original articles on a wide range of urological problems” (2010) instead of “scientific contributions in the field of clinical and basic research in urology” (2002).

**Figure 2:**
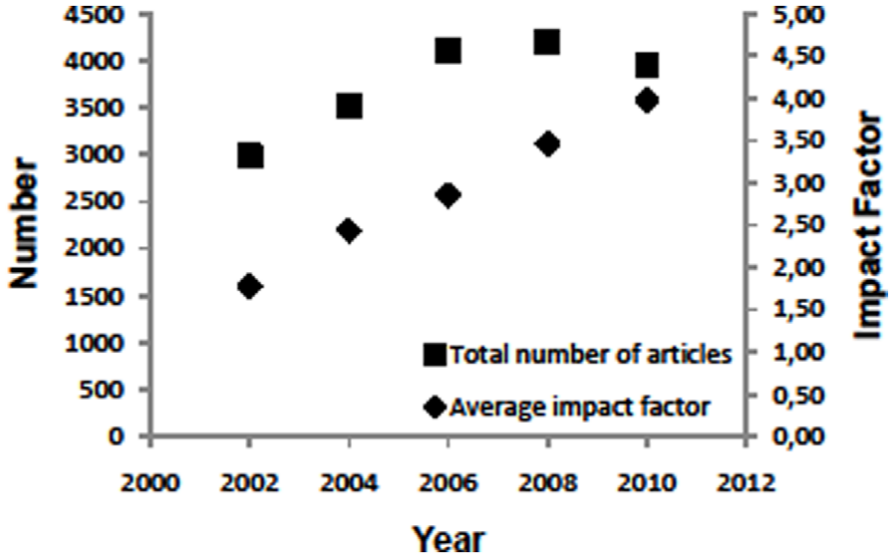
Total number of articles (from all journals) and average impact factor (averaged from all journals) from 2002–2010.

There was no significant correlation between the increase in the number of articles over the time period 2002 to 2010 and the impact factor, (Figure 2) which generally increased in all the journals except “Urology”, whose impact factor decreased slightly. The lack of significance was due to a biphasic behavior in the number of articles, increasing from 2002 to 2006 and maintaining a plateau until 2010. During 2002 to 2010 the average impact factor from all the journals increased steadily. In the journals with significant changes in the distribution of categorical weight from 2002 to 2010, the impact factor increased in “European Urology” from 1.8 to 8.8, in “World Journal of Urology” from 1.7 to 2.4 and in “Urologic Oncology” from 0.1 to 3.2. During the same time period, in those journals with no significant changes in the distribution of categorical weight, the impact factor in Journal of Urology was 3.0 increasing to 3.8, in “British Journal of Urology International” 1.6 increasing to 3.2 and in “Urology” 2.5 decreasing to 2.3 (Table 1).

**Table 1:** Correlation of impact factor (IF) 2002–2010 with percentage of articles in each category (clinical, experimental, review and commentarial).

	European Urology	Journal of Urology	British Journal of Urology International	Urology	World Journal of Urology	Urologic Oncology
	IF: 1.8–8.8	IF: 3.0–3.8	IF: 1.6–3.2	IF: 2.5–2.3	IF: 1.7–2.4	IF: 0.1–3.2
Clinical	r = −0.88	n.s.	n.s.	n.s.	n.s.	n.s.
	p = 0.05					
Experimental	n.s.	r = −0.89	n.s.	n.s.	n.s.	n.s.
		p = 0.04				
Review	r = −0.91	n.s.	n.s.	n.s.	n.s.	r = 0.92
	p = 0.03					p = 0.02
Commentarial	r = 0.89	n.s.	n.s.	n.s.	n.s.	n.s.
	p = 0.04					

n.s. = not significant, r = regression, p indicates significance value.

## Discussion

Management of urologic disease has undergone considerable progress during the period from 2002 to 2010. During this time period, in the four article categories - clinical, experimental, review and commentary - in the six journals, approximately 10% was devoted to experimental articles and 35% to clinical articles. However, journal specific differences were apparent. One journal significantly reduced the percentage of clinical articles (“European Urology”), whereas another journal significantly increased both the percentage of experimental and clinical articles (“World Journal of Urology”).

Since authors preferably submit their work to journals with a high impact factor, journals endeavor to adapt their editorial policy to increase the impact factor. Labanaris et al. recently provided evidence that journals publishing a high number of reviews or which include considerable amounts of letters, comments or other commentarial material are likely to increase their impact factors [5]. Since the journal volume is limited, increasing the percentage of non-original articles must necessarily diminish the printing space for original reports, which is counterproductive to exposing urologists to original research.

“European Urology” employed the publication strategy of decreasing the percentage of clinical articles in favor of articles with a commentarial content. The strategy was highly successful with regard to the journal’s impact factor, which increased from 1.8 in 2002 to 8.8 in 2010. However, due to the policy some universities have already adopted of excluding published commentarial material as a positive attribute in assessing an applicant’s scientific reputation or evaluating scientific output, this strategy to increase the impact factor may backfire and become obsolete. “Urologic Oncology” significantly decreased the percentage of commentarial articles from 2004 to 2010. Nevertheless, its impact factor was also enhanced from 0.1 in 2002 and 1.4 in 2004 to 3.2 (2010).

“World Journal of Urology” adopted a diametrically opposed strategy, increasing the percentage of original research articles. Considering the publication policies from 2004 to 2010 it is apparent that the increase in original research articles in “World Journal of Urology” was due to an increase in both clinical and experimental articles, at the cost of review articles. An increase in the impact factor from 1.7 in 2002 to 2.4 in 2010 took place, although this was much more modest, compared to “European Urology” and “Urologic Oncology”. In three journals, “The Journal of Urology”, “Urology”, and “British Journal of Urology International”, no significant trends in categorical percentage, indicating changes in their publication policies from 2002 to 2010 were apparent. Nevertheless, in these journals too, the impact factor, for the most part, increased.

The publication policy of these six journals was very heterogeneous and no clearly superior policy could be identified with regard to the general increase in impact from 2002 to 2010. The increase in impact cannot solely be attributed to an enlarged journal volume since the raise in the number of articles occurred during the time period 2002–2006 and then reached a plateau, while the average impact factor of all the journals continued to rise steadily to 2010 (Figure 2). Therefore, extending journal volume may be accompanied by an increase in impact, but a raise in the impact factor can also occur without volume extension.

This study was restricted to journals from the ISI-category “Urology and Nephrology”, excluding journals from other ISI-categories, e.g. “Oncology” or “Pharmacology and Pharmacy”, which also publish articles with urology related content. Based on this, we cannot generalize on whether the overall percentage of original articles concerning urological disease printed in all available journals has been expanded or not during the last years. It cannot be ruled out that the publication policies of pharmacologic and oncologic journals may differ from the publication policies of the urologic journals analyzed here.

Considering the importance of original research in fostering feasible therapeutic options and the various well-grounded appeals for exposing urologists to more research, it does seem reasonable to assume that there is room for expanding the percentage of articles concerned with original research. Only 10% of the articles in these six urologic journals were experimental and a doubling to 20%, as was apparent in “World Journal of Urology” was not disadvantageous to the impact factor. Experimental research is the basis for clinical research, which is preliminary to better disease management. Expanding the interface between experimental research and clinical research by increasing the percentage of experimental articles published could contribute to increasing the exposure of urologists to research, which presently is not optimal. Possibly, establishing new urologic journals, which chiefly reflect the molecular, biologic and pathophysiologic aspects of urological disorders may be helpful in increasing the number of original experimental publications. There is no indication that such policy would be detrimental to a journal’s impact factor.
